# HPV vaccination willingness and behavior among patients with cervical intraepithelial neoplasia in low-resource areas of Western China: a cross-sectional study

**DOI:** 10.3389/fpubh.2025.1708917

**Published:** 2026-01-22

**Authors:** Kefan Jiang, Yawen Shao, Huiling Wang, Fanghui Zhao, Li Su, Chengyun Li, Haitao Ma, Shumei Tuo, Ru Lin, Junling Wang

**Affiliations:** 1 School of Public Health, Lanzhou University, Lanzhou, Gansu, China; 2 Cervical Cancer Prevention and Treatment Center, Gansu Provincial Maternity and Child-care Hospital (Gansu Provincial Central Hospital), Lanzhou, Gansu, China; 3 Department of Cancer Epidemiology, National Cancer Center/National Clinical Research Center for Cancer/Cancer Hospital, Chinese Academy of Medical Sciences and Peking Union Medical College, Beijing, China

**Keywords:** attitude and practice, cervical intraepithelial neoplasia, human papillomavirus, knowledge, mediation analysis, vaccination behavior, vaccine willingness

## Abstract

**Background:**

Research on human papillomavirus (HPV) vaccination willingness and behavior among patients with cervical intraepithelial neoplasia (CIN) remains limited, although such evidence is essential for improving disease prevention.

**Methods:**

A knowledge, attitude, practice (KAP) questionnaire was administered to CIN patients aged 20–60 years in Gansu Province, China to explore HPV vaccine willingness and its influencing factors. Logistic regression identified demographic factors associated with vaccination willingness, including patients >45 years. Mediation analysis tested whether knowledge indirectly influenced willingness through attitude and practice. This study also compared the differences between willingness and actual behavior, and restricted cubic splines (RCS) assessed the age-behavior relationship.

**Results:**

Among 1,012 patients, 87.45% reported willingness to receive HPV vaccination, while only 27.96% had been vaccinated. Logistic regression showed that patients who were younger, lived in urban areas, had higher education and income, reported frequent sexual activity (>4 times/month), had more sexual partners, and achieved higher KAP scores demonstrated greater willingness (all *p* < 0.05). Mediation analysis showed that knowledge influenced vaccination willingness mainly through attitude and practice, especially attitude (*p* < 0.001), while a direct effect was observed only in the chain model. Among participants >45 years old (73.96% willing), those with high-grade squamous intraepithelial lesion (HSIL) were less likely to accept vaccination. RCS revealed a significant non-linear association between age and actual vaccination behavior (*p* < 0.001).

**Conclusion:**

Patients showed strong willingness to vaccinate, but the actual vaccination proportion was modest. Misconceptions that infection blocks vaccination may obscure the path to protection. It is recommended to highlight the role of HPV vaccination in preventing recurrence among older HSIL patients and extend the appropriate age of vaccination.

## Graphical abstract

**Figure:**
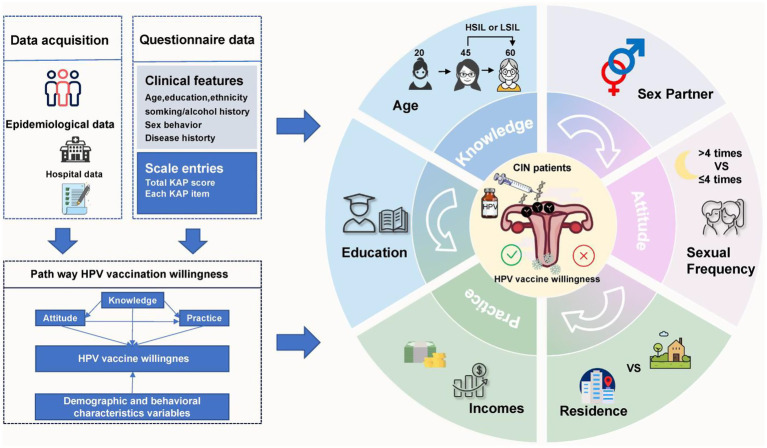
Willingness to receive HPV vaccination among CIN patients in low-resource settings.

## 1 Introduction

Cervical cancer is the fourth most common cancer threatening women’s health, representing an extremely serious disease burden (1). The shortage of high-quality medical resources and low public health awareness in low-resource settings make the current situation of cervical cancer prevention and control even less optimistic. Resource-constrained low- and middle-income countries account for nearly 85% of global cervical cancer cases, with China contributing about one quarter (2, 3), and with incidence rates in China’s resource-limited western regions remaining higher than in the east (4). Persistent high-risk HPV infection is the primary cause of cervical cancer and its precancerous lesions (CIN), both of which can be effectively prevented by HPV vaccination (5, 6). The incidence of cervical cancer is closely related to the effective management and prognosis of its precancerous lesions, making early prevention and intervention critical (7). CIN is classified by disease severity into low-grade squamous intraepithelial lesion (LSIL) and HSIL (8). LSIL represents an earlier-stage lesion that is more likely to regress spontaneously (9), whereas HSIL carries a substantially higher risk of progression to cervical cancer (10).

Most developing countries introduced the HPV vaccine rather late and failed to include it in their National Immunization Programs (NIPs) (11). Moreover, they lacked effective publicity, education, and vaccination services. Consequently, the vaccine coverage rate remains low, especially in low-resource settings of China, where the actual vaccination rate ranges from 0.06 to 2.02% (2, 12). This situation leaves a large number of women unvaccinated, increasing their probability of contracting HPV and subsequently developing precancerous cervical lesions. Nonetheless, evidence shows that CIN patients can still benefit from HPV vaccination (13). These benefits include preventing other HPV-type infections in patients with LSIL (14, 15), reducing the risk of disease progression or post-treatment recurrence in patients with HSIL (16–18), alleviating the psychological burden caused by repeated treatments and the adverse consequences of over-treatment (19), and even lowering the risk of progression to cervical cancer (20).

HPV vaccination willingness is an essential psychological driver of the act of getting vaccinated and reflects an individual’s personal motivation to receive the vaccine (21, 22). Most previous studies of this kind mainly concentrated on HPV vaccination willingness among women without cervical disease (23–25), while there is a relative paucity of studies in CIN patients. CIN patients’ perceptions and behaviors regarding vaccination may differ substantially from those of the general female population. A U.S. study showing that influenza experience increases both risk perception and vaccination behavior (26), some CIN patients also may develop greater risk awareness due to their disease experience and thus may hold more positive vaccine attitudes (27). However, others may believe the vaccine is intended only for those without disease and may doubt its necessity. And prior investigations have primarily focused on women of recommended vaccination age (28–30), while women aged 45 and older represent an often overlooked high-risk group. Evidence shows that age over 45 is a major risk factor for cervical cancer (31). Patients over 45 years old are beyond the recommended vaccination age (32), have a higher likelihood of cervical precancerous progression (31), and often frequently underestimate their personal risk due to decreased sexual activity (33). Therefore, more in-depth research is needed on the vaccination willingness of CIN patients to promote HPV vaccination and improve prognosis, including women aged 45 and above. Previous evidence have shown that demographic factors such as age, ethnicity, education, income, residence, and marital status, as well as behavioral factors including sexual activity, and HPV-related awareness and knowledge are associated with HPV vaccination willingness (34–36). Moreover, fear of disease progression has been shown to change vaccination willingness (37), suggesting that the differing progression risks of LSIL and HSIL may also affect CIN patients’ willingness, but evidence remains limited.

**Figure 1:**
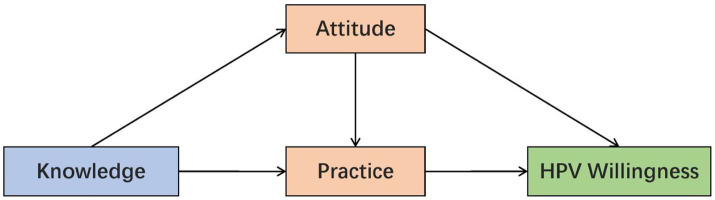
Conceptual framework of the hypothesized KAP mediation model for HPV vaccination willingness in CIN patients.

The KAP framework offers a widely used basis for understanding how health behaviors are formed (38). In this model, knowledge shapes attitudes, which then drive preventive practices (39). In the health behavior field, the KAP framework shows that greater HPV-related knowledge is associated with more favorable attitudes about HPV and its vaccine, which subsequently promote proactive prevention, treatment-seeking, and screening practices (40). Attitudes always mediate the relationship between knowledge and practice (41, 42). The KAP framework has also been shown to influence HPV vaccination willingness. The KAP model can comprehensively evaluate public perceptions and behaviors toward HPV and its vaccine, helping us understand the factors influencing HPV vaccination willingness (43). HPV knowledge influenced vaccine willingness both through attitude alone and through the sequential pathway from attitude to practice (44). Based on the above, we hypothesize that: (H1) knowledge indirectly influences willingness through attitude or practice (parallel mediation model); (H2) knowledge sequentially influences willingness via attitude and practice (Chain mediation model). We constructed a unified conceptual framework to assess the mediating roles of attitude and practice in the vaccination willingness of CIN patients, as shown in the Figure 1.

Actual vaccination behavior is defined as the objective action of whether an individual has received the vaccine (45). Although vaccine willingness is generally high, actual uptake remains low (46, 47), indicating that strong intention does not necessarily translate into action. Therefore, it is necessary to determine the actual vaccination rate among those who express willingness, which is essential for designing more targeted interventions. Meanwhile, given the wide age range of CIN patients and age often exhibits non-linear associations with various health behaviors or outcomes (48, 49), such as weight loss, this raises the question of whether age may likewise have a potential non-linear association with vaccination behavior.

Gansu Province is located in northwestern China and is classified as a low-resource area (50). The incidence and mortality rates of cervical cancer in this province are significantly higher than the national average, and its medical resources remain far more constrained than those in eastern regions, highlighting the necessity of strengthening the prevention and control of cervical cancer (2). In view of the deficiencies in prior studies, this study aims to: (1) explore HPV vaccination willingness among CIN patients in Gansu, along with its determinants and related reasons; (2) construct a mediation model to clarify the pathways through which knowledge, attitudes, and practices influence vaccination willingness; (3) identify key determinants of willingness among patients aged >45 years; and (4) investigate the discrepancy between vaccination willingness and actual vaccination behavior. This would provide a reference for the study of the willingness of high-risk populations in low-resource settings and help optimize vaccine promotion strategies.

## 2 Materials and methods

### 2.1 Study design and population

In this study, a cross-sectional survey was carried out. From January to September 2024, CIN outpatients diagnosed with cervical intraepithelial neoplasia at the Cervical Cancer Prevention and Treatment Center, Gansu Provincial Maternity and Child-care Hospital (Gansu Provincial Central Hospital), were recruited using a convenience sampling method.

Inclusion criteria: (1) were aged 20–60 years; (2) were diagnosed with CIN (including LSIL and HSIL) according to the 2020 WHO guidelines (51); (3) had a history of sexual activity; (4) had no cognitive impairment; (5) provided informed consent.

Exclusion criteria: (1) had a diagnosis of cervical cancer; (2) were pregnant; (3) had difficulties in comprehension or communication; (4) had severe physical or mental illness.

The age range of 20–60 years was chosen because women in early adulthood (around their early twenties) experience the first peak of HPV infection (52, 53), while the risks of cervical cancer and HPV infection remain relatively high around age 60 (52, 54). This range allowed us to capture CIN patients across both key risk periods and assess vaccination willingness before the incidence rises again.

This study was conducted in accordance with the Declaration of Helsinki. Ethical approval was obtained from the Ethics Committee of Gansu Provincial Maternity and Child-care Hospital (Gansu Provincial Central Hospital) (Approval No.: GSFY Ethics [95], September 25th, 2023). Informed consent was obtained from all participants prior to the survey.

### 2.2 Measures and instruments

The questionnaire consisted of three parts: baseline demographic and behavioral characteristics, HPV and its vaccine KAP scale, and the HPV vaccination willingness and behavior questionnaire. The survey questionnaire and informed consent form are provided in Supplementary file S1.

#### 2.2.1 Baseline demographic and behavioral characteristics

General social demographic information. A total of 22 entries covering age, ethnicity, residence, religion, education level, occupation, healthcare payment methods, total monthly household income, history of smoking, history of alcohol consumption, marital status, age at first sexual intercourse, monthly sexual intercourse frequencies, 6-month partner count, number of all sexual partners, condom use, contraceptive use, frequency of pregnancies, number of deliveries, disease severity, history of frequent gynecological infections, and cervical cancer family history.

#### 2.2.2 HPV and its vaccine KAP scale

Based on the KAP theoretical framework, the initial item pool was constructed after preliminary qualitative discussions and a literature review, referencing recent WHO HPV vaccination guidance (55) and prior HPV-related KAP studies (24, 28, 56).

Three senior gynecologists and five epidemiology experts from Lanzhou University reviewed all preliminary items. Based on their suggestions, one irrelevant behavior item (“Will you adopt protective measures such as condom use in the future?”) was removed and one new behavior item (“Would you recommend the HPV vaccine to your friends?”) was added. Wording revisions were made to ensure item relevance, clarity, and completeness.

Following the requirement of having at least 10 participants per item of the largest dimension (41), 133 CIN patients were recruited for the pre-survey. Participants were encouraged to provide feedback on items that were unclear or difficult to understand. The results showed that the Cronbach’s *α* coefficients for the HPV and its vaccine knowledge, attitude, practice, and KAP total scales were 0.816, 0.905, 0.703, and 0.923, respectively, and the corresponding test–retest reliabilities were 0.950, 0.969, 0.879, and 0.971. Exploratory factor analysis of the total scale revealed a Kaiser-Meyer-Olkin (KMO) value of 0.812 (>0.70) and a Bartlett’s test of sphericity χ^2^ value = 1,526.613 (*p* < 0.001). The KMO measure assesses sampling adequacy for factor analysis and is commonly used to evaluate whether data are suitable for validity testing (57). The above results indicate that the questionnaire has good reliability and validity and can be applied in this research study. The final scale is structured around three primary dimensions and encompasses 42 items, including 26 knowledge items, 12 attitude items, and 4 practice items. For each item, the response options are “yes,” “not sure,” and “no,” scoring 3, 2, and 1, respectively.

#### 2.2.3 Willingness and current status of HPV vaccination questionnaire

This section contains a total of 4 entries. The question for assessing willingness to be vaccinated is “Are you willing to be vaccinated against HPV?,” with the options “willing” and “not willing” being scored 1 and 0, respectively. The question for assessing the actual status of vaccination was “Have you been vaccinated against HPV?,” with “yes” and “no” options scored 1 and 0, respectively. In this study, all previously received HPV vaccination is hereafter referred to as actual vaccination behavior (including HPV vaccinations received before age 45 among participants who were in the >45-year group). The other two multiple-choice questions were “What are the reasons why you are willing to receive HPV vaccination?” and “What are the reasons why you are not willing to receive HPV vaccination?”

### 2.3 Sample size

The study was a cross-sectional survey and the sample size was calculated using the following formula:

$$N=\frac{\mu_{1-\alpha /2}^{2}p(1-p)}{\delta^{2}}$$

According to the results of a meta-analysis, the willingness of women in mainland China to be vaccinated against HPV was 76.12% (58) (*p* = 0.7612); Using 1–α/2 = 1.96 (α = 0.05, two-sided), δ = 0.03 (the allowable margin of error), the minimum sample size for this study was calculated to be 776. Considering 10% invalid questionnaires, the final sample size was estimated at 862.

### 2.4 Data quality control

All questionnaires were completed face-to-face by uniformly trained investigators and patients. For topics that are difficult for patients to understand, investigators are required to provide detailed explanations and guidance so as to ensure that the patients fully understand and accurately answer the questionnaires. After completing the questionnaire, the investigator immediately collects the questionnaire and conducts a comprehensive check to ensure the completeness, objectivity, and logic of the content. This data collection method aims to minimize errors and biases during the survey process and ensure the quality of the obtained data.

### 2.5 Statistical analysis

This study utilized Statistical Package for the Social Sciences (SPSS) 26.0 and R 4.4.3 for data analysis. Once the data collection for this study was completed, it was thoroughly examined for any missing data, and all the data could be used for subsequent analysis. First, the normality of continuous variables was assessed using the Shapiro–Wilk test. Results indicated that none of the continuous variables followed a normal distribution. Therefore, they were presented as median (interquartile range, IQR), and group comparisons were performed using the Mann–Whitney U test for independent samples and Z values were reported. Categorical variables were expressed as frequency (N) and percentage (%) and between-group differences were assessed using the chi-square test and chi-square values were reported. Effect sizes were reported using r for non-parametric tests and Cramer’s V for chi-square tests. Second, RCS is a flexible regression method used to model potential non-linear relationships between continuous predictors and outcomes (48). To examine the potential non-linear association between age and actual HPV vaccination behavior among participants with vaccination willingness, we applied a multivariable logistic regression model incorporating RCS functions for age. Using the median age of 39 years as reference, odds ratios (ORs) and 95% confidence intervals (CIs) were estimated after adjusting for relevant confounders. Moreover, we constructed three models exploring the associations of KAP scores with HPV vaccination intentions by stepwise adjustment of covariates, respectively.

To investigate the mediating roles of KAP in the association between predictors and HPV vaccination willingness among CIN patients, we conducted both parallel and chain mediation analyses using the *mediation* package in R under the causal mediation analysis (CMA) framework (59). This framework estimates natural direct and indirect effects under a counterfactual causal inference approach without relying on traditional linear path coefficients (60–62). It is particularly suitable for nonlinear models with binary outcomes, such as logistic mediation models commonly applied in health behavior research (63, 64). Given that the outcome (HPV vaccination willingness) was binary and the KAP variables were continuous, mediator models were estimated using linear regression, while the outcome models were estimated using logistic regression (65). Nonparametric bootstrapping with 5,000 resamples was applied to derive 95% confidence intervals for direct and indirect effects. In the parallel mediation model, attitude and practice were considered as independent mediators, whereas the chain mediation model tested a sequential pathway from knowledge to attitude to practice to vaccination willingness. Additionally, we used the *lavaan* package in R to estimate standardized path coefficients under linear assumptions. These coefficients are presented only to illustrate the direction and strength of associations, rather than for causal inference.

All analyses were considered statistically significant at two-sided *p* < 0.05, and the model construction code is provided in Supplementary file S2.

## 3 Results

### 3.1 Demographic characteristics of the total CIN patients

A total of 1,050 questionnaires were distributed in this study, and 1,012 valid questionnaires were returned, with a validity rate of 96.38%. The median age was 40.5 years, 62.55% had a high school or secondary school education or higher, 21.44% were farmers. 55.43% had a total monthly household income of RMB 5,000 and above. Regarding sexual behavior, 84.88% patients had one sexual partner in the past 6 months, and 62.45% of all patients had one sexual partner. In addition, 1,004 participants (99.21%) indicated no family history of cervical cancer. Median knowledge, attitude, and practice scores were 68, 32, and 12, respectively. Among all participants, 885 (87.45%) were willing to receive HPV vaccination and 127 (12.55%) were unwilling. Participants aged ≤45 years showed markedly higher willingness compared with those >45 years (*p* < 0.001). Urban residents were far more willing than rural residents (78.42% vs. 21.58%, *p* < 0.001). Higher education was strongly associated with willingness (*p* < 0.001). Married women were more willing than divorced/widowed women (*p* = 0.048). Unemployed and farmer participants showed lower willingness than those in other occupations (*p* < 0.001). Participants with a monthly household income ≥5,000 yuan were more willing than those earning <3,000 yuan (*p* < 0.001). Lower willingness was observed among women with early sexual debut (20 years), lower sexual frequency, no partner in the past 6 months, only one lifetime partner, no condom or contraceptive use, higher parity, or HSIL diagnosis (all *p* < 0.05). Women with frequent gynecological infections also showed lower willingness (*p* = 0.031). Knowledge, attitude, and practice scores were consistently higher in the willingness group than in the unwillingness group (all *p* < 0.001). See Table 1 for details.

**Table 1:** Baseline demographic and behavioral characteristics of CIN patients and univariable analysis of HPV vaccination willingness.

Demographic information	*N* = 1,012	Willingness to vaccinate, M (IQR)/*N* (%)		*Z/χ^2^*	*p* value	Effect sizes
		Willingness group *N* = 885	Unwillingness group *N* = 127			
Age, (years)	40.5 (IQR: 34.25–49)	39 (IQR: 34–47)	52 (IQR: 49–55)	−9.280	<0.001	0.292
≤45 years	674 (66.60)	635 (71.75)	39 (30.71)	80.045	<0.001	
>45 years	338 (33.40)	250 (28.25)	88 (69.29)			
Ethnicity				0.143	0.706	0.012
Han	948 (93.68)	830 (93.79)	118 (92.91)			
Ethnic Minority	64 (6.32)	55 (6.21)	9 (7.09)			
Residence				111.433	<0.001	0.332
City	737 (72.83)	694 (78.42)	43 (33.86)			
Countryside	275 (27.17)	191 (21.58)	84 (66.14)			
Religions				0.849	0.357	0.029
Yes	104 (10.28)	88 (9.94)	16 (12.60)			
No	908 (89.72)	797 (90.06)	111 (87.40)			
Educational level				155.006	<0.001	0.391
Primary school and lower	167 (16.50)	100 (11.30)	67 (52.76)			
Junior high school	212 (20.95)	180 (20.34)	32 (25.19)			
High school or secondary school education and higher	633 (62.55)	605 (68.36)	28 (22.05)			
Marital status				6.066	0.048	0.077
Single	34 (3.36)	34 (3.84)	0 (0)			
Married	920 (90.91)	803 (90.74)	117 (92.13)			
Divorced and widowed	58 (5.73)	48 (5.42)	10 (7.87)			
Occupation				114.477	<0.001	0.348
Unemployed	153 (15.12)	123 (13.90)	30 (23.62)			
Farmer	217 (21.44)	149 (16.83)	68 (53.54)			
Others	642 (63.44)	613 (69.27)	29 (22.84)			
Healthcare payment methods				51.276	<0.001	0.225
Urban and Rural Resident Basic Medical Insurance	236 (23.32)	188 (21.24)	48 (37.79)			
Employee Basic Medical Insurance	379 (37.45)	367 (41.47)	12 (9.45)			
Commercial Health Insurance	4 (0.40)	4 (0.45)	0 (0.00)			
Self-payment	393 (38.83)	326 (36.84)	67 (52.76)			
Total monthly household income, (yuan)				173.125	<0.001	0.413
<3,000	162 (16.01)	94 (10.62)	68 (53.55)			
3,000–5,000	289 (28.56)	248 (28.02)	41 (32.28)			
5,000 and above	561 (55.43)	543 (61.36)	18 (14.17)			
Smoking				1.684	0.300	0.041
Yes	57 (5.63)	53 (5.99)	4 (3.15)			
No	955 (94.37)	832 (94.01)	123 (96.85)			
Alcohol consumption				13.897	<0.001	0.117
Yes	321 (31.72)	299 (33.79)	22 (17.32)			
No	691 (68.28)	586 (66.21)	105 (82.68)			
Age at first sexual intercourse, (years)				5.789	0.122	0.076
<15 years	8 (0.79)	6 (0.67)	2 (1.57)			
15–20 years	229 (22.63)	193 (21.82)	36 (28.35)			
20–25	562 (55.53)	492 (55.60)	70 (55.12)			
≥25	213 (21.05)	194 (21.91)	19 (14.96)			
Sexual intercourse frequencies, (times/month)				7.314	0.007	0.085
≤4	524 (51.78)	444 (50.17)	80 (62.99)			
>4	488 (48.22)	441 (49.83)	47 (37.01)			
6-month partner count, (persons)				6.465	0.039	0.080
0	122 (12.06)	98 (11.07)	24 (18.90)			
1	859 (84.88)	760 (85.88)	99 (77.95)			
2 and above	31 (3.06)	27 (3.05)	4 (3.15)			
Number of all sexual partners, (persons)				31.856	<0.001	0.177
1	632 (62.45)	524 (59.21)	108 (85.04)			
2	228 (22.53)	215 (24.29)	13 (10.24)			
3 and above	152 (15.02)	146 (16.50)	6 (4.72)			
Condom use				88.681	<0.001	0.296
Always (each time or often)	252 (24.90)	242 (27.34)	10 (7.87)			
Occasionally	351 (34.68)	334 (37.74)	17 (13.39)			
Never	409 (40.42)	309 (34.92)	100 (78.74)			
Contraceptive use				40.590	<0.001	0.200
Always (each time or often)	16 (1.58)	15 (1.69)	1 (0.78)			
Occasionally	314 (31.03)	305 (34.46)	9 (7.09)			
Never	682 (67.39)	565 (63.83)	117 (92.13)			
Frequency of pregnancies	2 (IQR: 2–4)	2 (IQR: 1–3)	3 (IQR: 2–4)	−3.251	<0.001	0.102
Number of deliveries	1 (IQR: 1–2)	1 (IQR: 1–2)	2 (IQR: 2–3)	−7.812	<0.001	0.246
Disease severity				15.462	<0.001	0.124
LSIL	691 (68.28)	614 (69.38)	77 (60.63)			
HSIL	321 (31.72)	271 (30.62)	50 (39.37)			
History of frequent gynecological infections, (≥3 times/year)				4.670	0.031	0.068
Yes	596 (58.89)	510 (57.63)	86 (67.72)			
No	416 (41.11)	375 (42.37)	41 (32.28)			
Cervical cancer family history				1.157	0.282	0.034
Yes	8 (0.79)	8 (0.90)	0 (0.00)			
No	1,004 (99.21)	877 (99.10)	127 (100.00)			
Knowledge score	68 (IQR: 60–73)	69 (IQR: 63–73)	52 (IQR: 52–54)	−16.066	<0.001	0.505
Attitude score	32 (IQR: 30–34)	33 (IQR: 31–34)	23 (IQR: 22–27)	−16.777	<0.001	0.527
Practice score	12 (IQR: 10–12)	12 (IQR: 11–12)	6 (IQR: 4–8)	−18.640	<0.001	0.586
Total	1012 (100.00)	885 (100.00)	127 (100.00)			

Continuous variables are presented as M (IQR), where M is the median and IQR is the interquartile range (25th-75th percentiles). Categorical variables are presented as N (%) indicating frequency and percentage. N, number of participants; Z, Z values for Mann–Whitney U test; χ^2^, chi-square values for categorical variables; OR, odds ratio; 95% CI, 95% confidence interval.

### 3.2 Differences between HPV vaccination willingness and actual vaccination behavior among CIN patients

**Figure 2:**
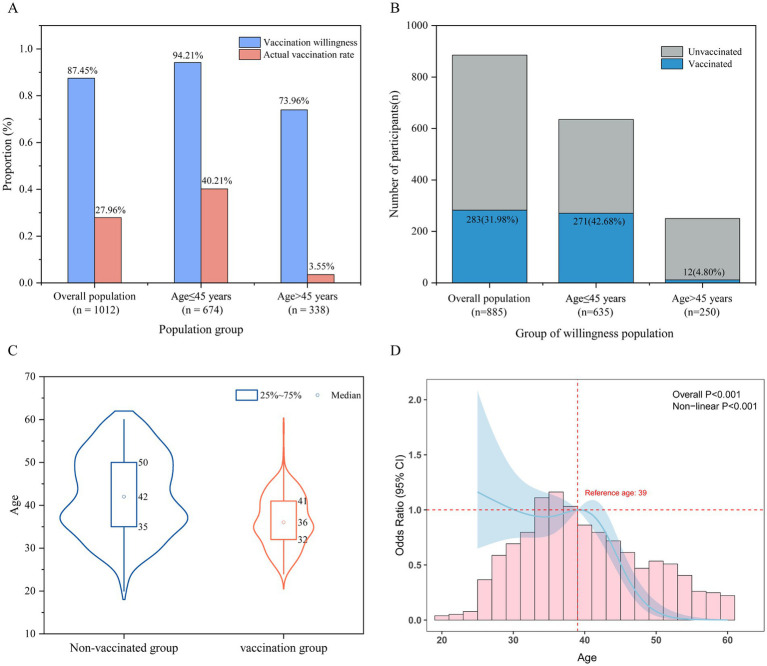
The relationship between CIN patients’ willingness to receive HPV vaccination and their actual vaccination behavior. **(A)** Comparison of vaccination willingness and actual (≤45 years) or precious (45 years) vaccination proportion in the overall population and by age group; **(B)** Proportion of vaccinated and unvaccinated participants within the willingness group, stratified by age group; **(C)** Age distribution of vaccinated and unvaccinated participants in the willingness group; **(D)** RCS curve showing the odds ratio (OR) for actual (≤45 years) or past (45 years) vaccination by age among participants with willingness; shaded areas indicate 95% confidence intervals.

As shown in Figure 2A, the actual vaccination proportion of total CIN patients was 27.96%. In the ≤45-year-group, the willingness rate was 94.21%, and the actual vaccination proportion was 40.21%. In the >45-year group, the willingness rate was 73.96%, but the vaccination proportion was only 3.55%. Among 885 participants with vaccination willingness, the actual vaccination proportion was 31.98% (see Figure 2A). Substantial differences in vaccination coverage were observed across age groups: 42.68% in the ≤45-year-group versus 4.80% in the >45-year-group (see Figure 2B). As shown in Figure 2C, age distribution differed significantly between vaccinated and unvaccinated participants within the willingness group (*p* < 0.001), with unvaccinated individuals being older on average and having a wider age range (median: 42 years vs. 36 years). In the willingness group, RCS analysis revealed a significant non-linear association between age and actual HPV vaccination behavior (overall χ^2^ = 48.94, df = 3, *p* < 0.001; non-linear χ^2^ = 27.31, df = 2, *p* < 0.001). Compared to the reference age of 39 years, the likelihood of vaccination peaked at approximately 25 years of age. The vaccination proportion among women aged 30–40 years temporarily declined before rebounding, showing a slight decrease from ages 30 to 35 followed by a rebound between ages 35 and 40. The proportion declined markedly after the age of 40. The model demonstrated acceptable discrimination (C-index = 0.675, likelihood ratio test *p* < 0.001) (see Figure 2D).

### 3.3 Analysis of HPV infection and its vaccine KAP items

**Figure 3:**
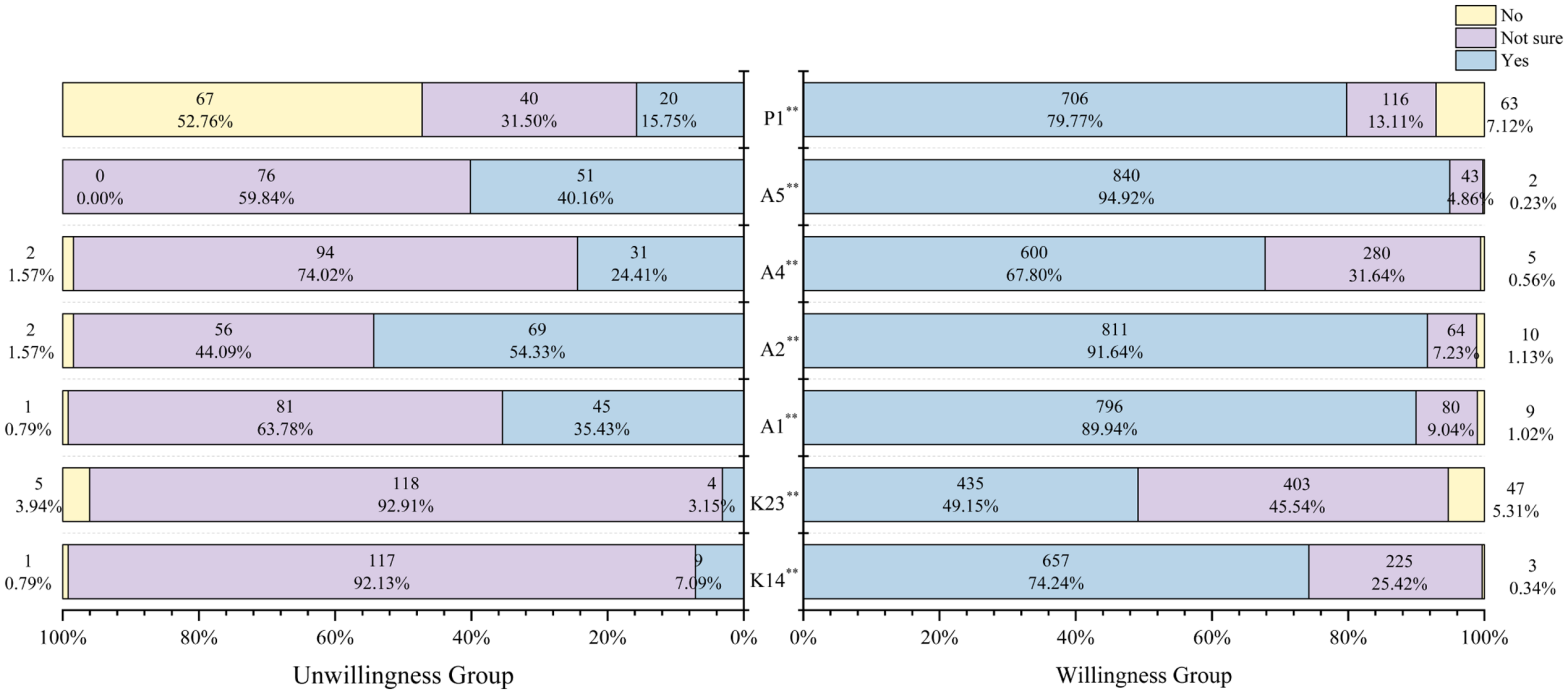
Analysis of selected entries of the KAP scale for patients with precancerous cervical lesions. (1) Knowledge items: K14: can HPV vaccine effectively prevent cervical cancer? K23: Can people who are infected with HPV also receive the HPV vaccine? (2) Attitude items: A1: are you worried about getting infected with HPV again after being cured? A2: are you worried about your disease coming back after treatment? A4: do you think HPV infection can be cured? A5: After treatment, would you be willing to have regular HPV testing to screen for cervical cancer? (3) Practice items: P1: would you be proactive in learning about treatment for HPV infection? (**: *p* < 0.001).

In view of the special characteristics of CIN patients, a total of seven representative and important entries from the KAP scale were selected for analysis in this study. Compared to the willingness group, the unwillingness group demonstrated significantly lower knowledge scores and deficiencies in key KAP entries, highlighting a substantial cognitive gap. In terms of knowledge, the willingness group’s responses to the questions “Can HPV vaccine effectively prevent cervical cancer?” (74.24% vs. 7.09%) and “Can people infected with HPV also receive HPV vaccine?” (49.15% vs.3.15%) were answered correctly at a much higher rate compared to the unwillingness group’s responses (*p* < 0.001). In terms of attitude, the willingness group responded to the questions “Are you worried about re-infection with HPV after cure?” (89.94% vs.35.43%), “Are you worried about recurrence of your disease after treatment?” (91.64% vs.54.33%), “Do you think you can be cured after HPV infection?” (67.80% vs.24.41%) and “After treatment, would you be willing to have regular HPV tests to screen for cervical cancer?” (94.92% vs.40.16%) had much higher correct response rates than the unwillingness group (*p* < 0.001). Regarding practice, the willingness group had much higher correct response rates to the question “Would you take the initiative to learn about treating HPV infection?” (79.77%) compared to the unwillingness group (15.75%) (*p* < 0.001) (see Figure 3).

### 3.4 Multifactor regression analysis of HPV vaccination willingness among total population

With HPV vaccination willingness as the dependent variable to be analyzed, variables identified as significant in the univariate analysis were selected as independent variables (see Table 1). As shown in Table 2, the variable screening method used was the stepwise backward method and we used it to construct the binary logistic regression model. The analysis showed that older age (OR = 0.947, 95%CI: 0.926–0.968, *p* < 0.001) and residence in rural areas (OR = 0.546, 95%CI: 0.365–0.816, *p* < 0.001) were negative factors influencing HPV vaccination willingness among CIN patients. The positive factors for HPV vaccination willingness included: education level of high school or above (OR = 2.996, 95%CI: 1.832–4.902, *p* < 0.001), total monthly household income of 3,000–5,000 yuan (OR = 2.914, 95%CI: 1.932–4.394, *p* < 0.001) as well as more than 5,000 yuan (OR = 6.812, 95%CI: 4.058–11.435, *p* < 0.001), monthly sexual frequency greater than 4 times (OR = 1.460, 95%CI: 1.012–2.106, *p* = 0.043), and having 2 sexual partners (OR = 1.828, 95%CI: 1.091–3.061, *p* = 0.022) (see Table 2).

**Table 2:** Multifactor regression analysis of HPV vaccination willingness among total population.

Independent variable	β	Waldχ^2^	OR (95%CI)	*p v*alue
Age, (years)	−0.055	23.228	0.947 (0.926–0.968)	<0.001
Educational level				
Primary school and lower			Ref.	
Junior high school	0.855	14.613	2.352 (1.517–3.464)	<0.001
High school or secondary school education or higher	1.097	19.096	2.996 (1.832–4.902)	<0.001
Residence				
City			Ref.	
Countryside	−0.606	8.726	0.546 (0.365–0.816)	0.003
Total monthly household income, (yuan)				
<3,000			Ref.	
3,000–5,000	1.069	26.019	2.914 (1.932–4.394)	<0.001
5,000 and above	1.919	52.716	6.812 (4.058–11.435)	<0.001
Sexual intercourse frequencies (times/month)				
≤4			Ref.	
>4	0.378	4.100	1.460 (1.012–2.106)	0.043
Number of all sexual partners, (persons)				
1			Ref.	
2	0.603	5.252	1.828 (1.091–3.061)	0.022
3 and above	0.604	2.813	1.829 (0.903–3.705)	0.094
Constant	1.875	8.525	–	0.004

*β*, regression coefficient; OR, odds ratio; CI, confidence interval; Wald χ^2^, Wald chi-square statistic; Ref., reference category.

### 3.5 Association between KAP scores and HPV vaccination willingness

As shown in Table 3, the results from the three regression models constructed in this study showed that knowledge and attitude were correlated with practice and willingness to vaccinate, with the results from model 3 showing that the higher the level of knowledge (OR = 1.086, 95%CI: 1.003–1.176, *p* = 0.042), the more positive attitude (OR = 1.713, 95%CI: 1.422–2.063, *p* < 0.001), and the more proactive practice (OR = 2.049, 95%CI: 1.609–2.608, *p* < 0.001) the higher the willingness of HPV vaccination in CIN patients (see Table 3).

**Table 3:** Association between KAP scores and HPV vaccination willingness.

Variables	Model 1		Model 2		Model 3	
	OR (95% CI)	*p v*alue	OR (95%CI)	*p v*alue	OR (95%CI)	*p v*alue
Knowledge	1.073 (1.002–1.148)	0.044	1.082 (1.003–1.167)	0.041	1.086 (1.003–1.176)	0.042
Attitude	1.562 (1.343–1.816)	<0.001	1.639 (1.387–1.937)	<0.001	1.713 (1.422–2.063)	<0.001
Practice	1.726 (1.441–2.067)	<0.001	1.787 (1.460–2.189)	<0.001	2.049 (1.609–2.608)	<0.001

OR, odds ratio; CI, confidence interval.

Model 1: No covariates controlled.

Model 2: Controlled for sociodemographic variables, including age, ethnicity, current residence, religious, education level, occupation, marital status, method of medical payment, and total monthly household income.

Model 3: Controlled for all covariates, additionally including smoking, alcohol consumption, age at first sexual intercourse, frequency of sexual activity, number of sexual partners in the past 6 months, total number of sexual partners, condom usage frequency, oral contraceptive usage frequency, number of pregnancies, number of deliveries, disease severity, history of frequent gynecological inflammation, and family history of cervical cancer.

### 3.6 Mediating effects of KAP on HPV vaccination willingness

**Figure 4:**
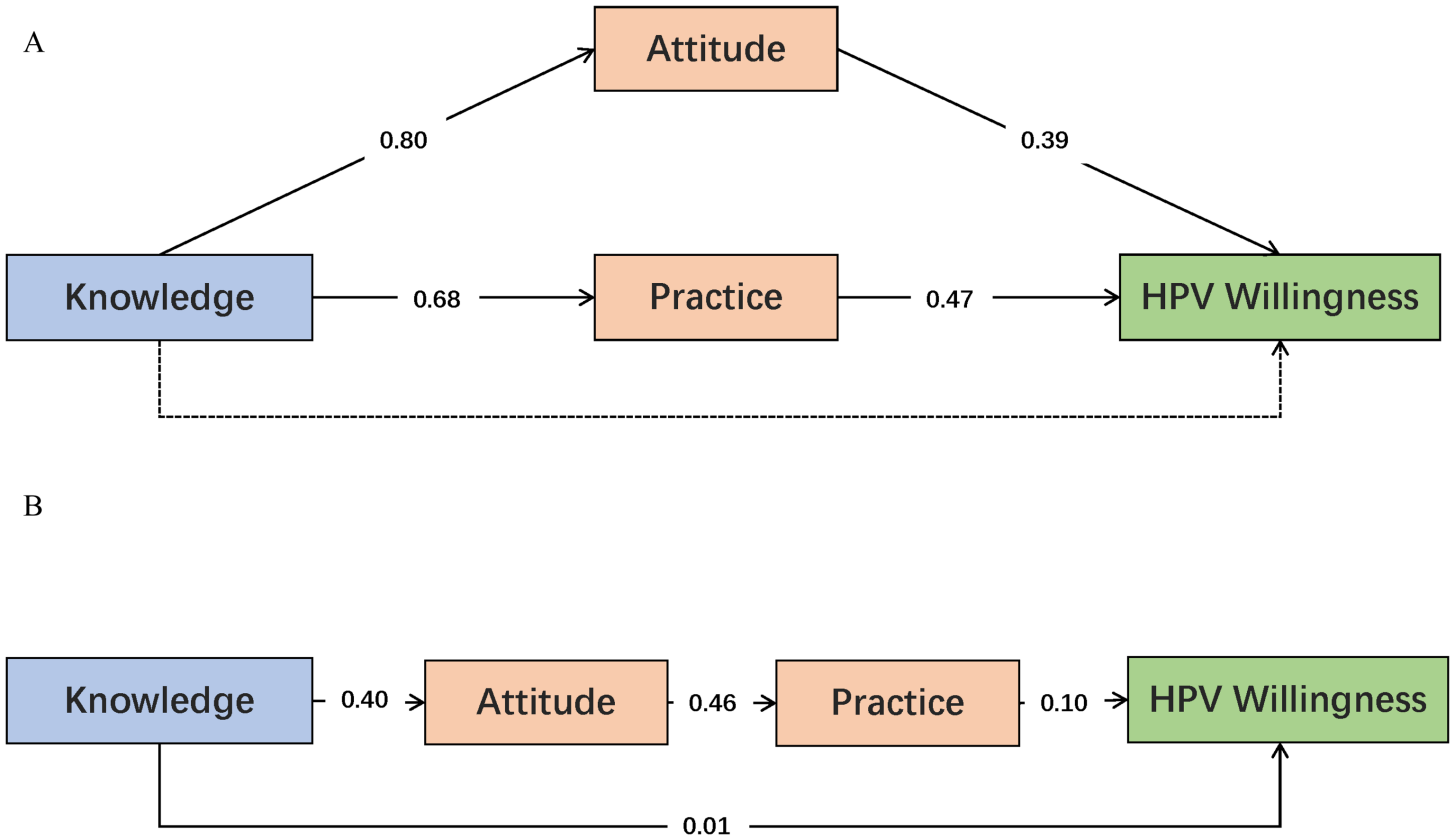
Mediation analysis results for KAP on HPV vaccination willingness among CIN patients. **(A)** Parallel mediation model of knowledge, attitude, and practice on HPV vaccination willingness. **(B)** Chain mediation model of knowledge, attitude, and practice on HPV vaccination willingness. Solid lines indicate statistically significant direct or indirect effects, while dashed lines represent non-significant effects. The standardized path coefficients shown in this figure are presented only to illustrate the direction and strength of associations, rather than for causal inference.

Based on the KAP theoretical framework, after controlling for demographic variables including age, education level, place of residence, monthly household income, monthly frequency of sexual activity, and number of sexual partners, this study constructed a parallel mediation model and a chain mediation model to examine the indirect effects and mediating roles of knowledge, attitude, and practice on HPV vaccination willingness. The detailed mediation results are presented in Supplementary Table S1. In the parallel mediation model (see Figure 4A), the indirect effect of knowledge on HPV vaccination willingness through attitude was significant (*p* < 0.001), with a mediation proportion of 68.37%. Knowledge also exerted a significant indirect effect on HPV vaccination willingness through practice (*p* < 0.001), with a mediation proportion of 50.99%. However, the direct effect of knowledge on HPV vaccination willingness was not significant (*p* = 0.137), suggesting that knowledge primarily influences vaccination willingness indirectly via attitude and practice. In the chain mediation model (see Figure 4B), knowledge exerted both a direct effect (*p* < 0.001) on HPV vaccination willingness and an indirect effect via the chained path from attitude to practice, with a mediation proportion of 31.63%. Thus, attitude served as the primary mediator between knowledge and HPV vaccination willingness, while practice also played a mediating role (see Figure 4B).

### 3.7 Multifactor regression analysis of HPV vaccination willingness among patients aged >45 years

**Figure 5:**
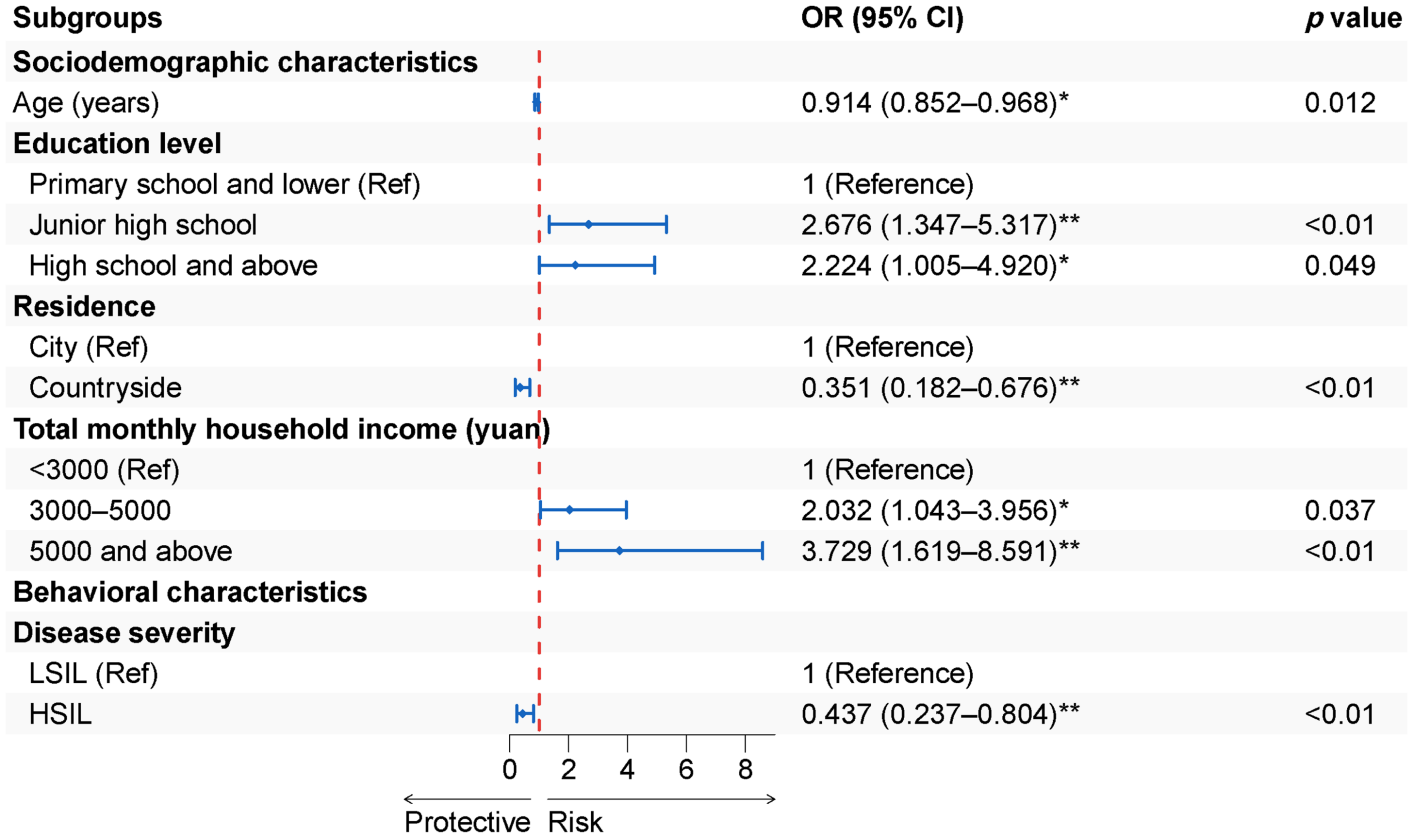
General demographic and behavioral characteristics factors associated with HPV vaccination willingness of >45-year-group.

For patients aged >45 years, HPV vaccination willingness was taken as the dependent variable, with independent variables selected based on significance in the univariate analysis (see Supplementary Table S2). In this study, we also specifically explored the factors influencing HPV vaccination willingness among patients aged >45 years. Using the stepwise backward method, we constructed a binary logistic regression model. As shown in Figure 5, the analysis showed that older age (OR = 0.914, 95%CI: 0.852–0.980, *p =* 0.012), residence in rural areas (OR = 0.351, 95%CI: 0.182–0.676, *p* < 0.001) and disease severity of HSIL (OR = 0.437, 95%CI: 0.237–0.804, *p <* 0.001) were negative factors influencing HPV vaccination willingness among CIN patients aged >45 years. The positive factors for HPV vaccination willingness in the >45-year group included: education level of high school or above (OR = 2.224, 95%CI: 1.005–4.920, *p* = 0.049), total monthly household income of 3,000–5,000 yuan (OR = 2.032, 95%CI: 1.043–3.956, *p* = 0.037) as well as more than 5,000 yuan (OR = 3.729, 95%CI: 1.619–8.591, *p* = 0.002) (see Figure 5). In patients aged >45 years, regression models further indicated that attitude and practice were significantly associated with vaccination willingness, whereas knowledge showed no significant association (*p* > 0.05) (see Supplementary Table S3).

### 3.8 Analysis of reasons for willingness to receive HPV vaccination

**Figure 6:**
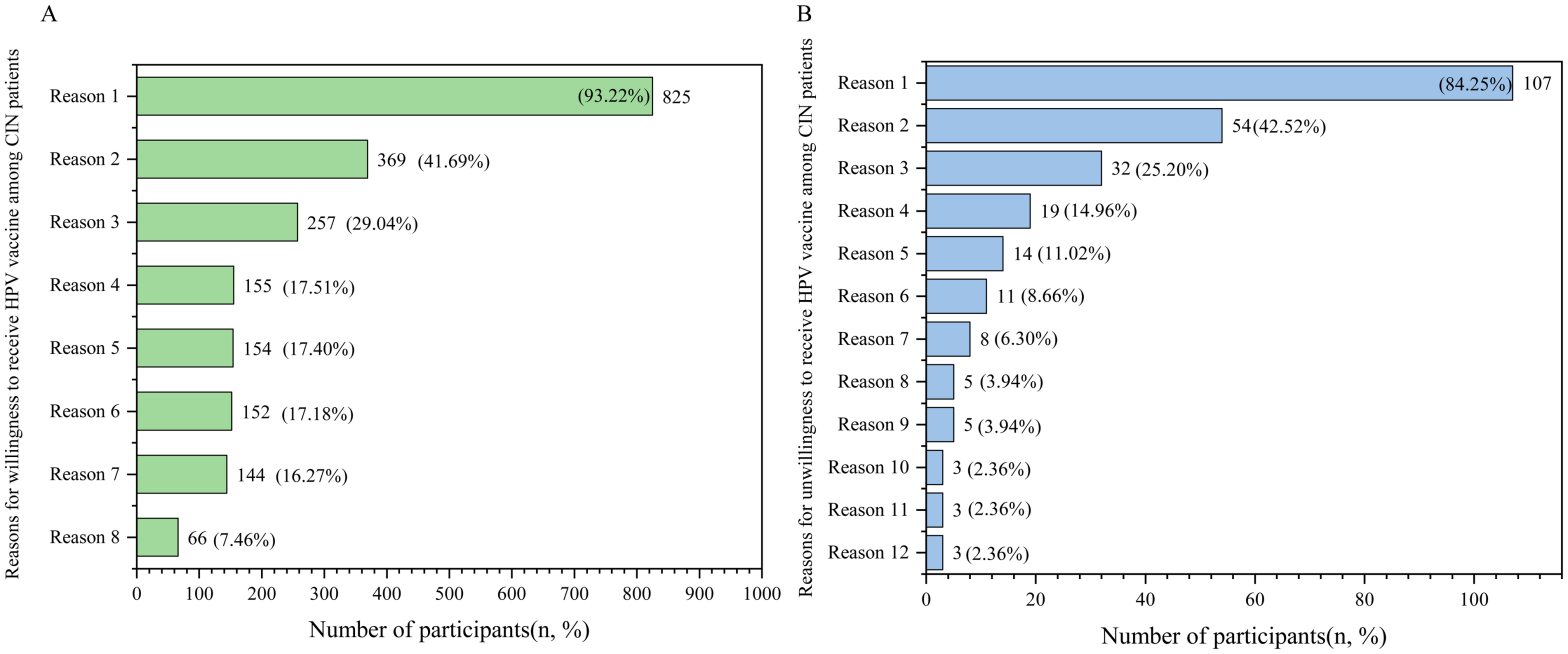
Reasons for willingness and unwillingness to receive HPV vaccine among total CIN patients. **(A)** Reasons for willingness to receive HPV vaccine among CIN patients: Reason 1: preventing cervical cancer and preventing the disease from worsening. Reason 2: you are already infected with HPV and worried about recurrence after treatment. Reason 3: you are worried about your partners or loved ones being infected with HPV-related diseases. Reason 4: the vaccine is safe. Reason 5: physician advocacy influence. Reason 6: vaccines are very effective. Reason 7: HPV vaccination for family and friends is highly recommended. Reason 8: community health service centers and other institutions’ HPV educational activities. **(B)** Reasons for unwillingness to receive HPV vaccine among CIN patients: Reason 1: not knowing about HPV and HPV vaccine. Reason 2: you are beyond the age of vaccination. Reason 3: you have already been infected with HPV and are worrying about the lack of usefulness in the follow-up. Reason 4: vaccines are expensive. Reason 5: concern about the safety of the vaccine. Reason 6: concern about the side effects of vaccines. Reason 7: concern about the effectiveness of vaccines. Reason 8: difficulty in making vaccine appointments. Reason 9: the local doctor said it’s not useful to inoculate against the infection. Reason 10: family members do not support their own vaccination. Reason 11: cumbersome vaccination procedures. Reason 12: reproductive needs.

As shown in Figure 6, the top three reasons for the willingness group to receive HPV vaccine were prevention of cervical cancer and combating the progression of the disease, accounting for 93.22%, having already been infected with HPV and worrying about recurrence after treatment, accounting for 41.69%, and worrying about their partners or loved ones being infected with HPV-related diseases, accounting for 29.04%. Additionally, 17.40% were willing to be vaccinated due to the influence of physicians’ advocacy. As for those who are not willing to be vaccinated, the top three reasons are lack of knowledge about HPV and its vaccine, accounting for 84.25%, believing that they are beyond the age of vaccination, accounting for 42.52%, and having already been infected with HPV and worrying about the lack of usefulness in the follow-up, accounting for 25.20% (see Figure 6).

## 4 Discussion

This study integrates the KAP framework with mediation analysis to systematically assess HPV vaccination willingness and the pathways through which knowledge, attitude, and practice influence this willingness among CIN patients in low-resource settings in China. We also compared KAP scores and the reasons for vaccination between the willingness and unwillingness groups, and examined the gap between vaccination willingness and actual vaccination behavior across the overall sample, the willingness group, and different age groups.

### 4.1 HPV vaccination willingness and actual vaccination behavior in CIN patients

In our study, 87.45% of CIN patients were willing to vaccinate, a level higher than that reported in the other general female populations in China (28, 29, 35, 66–69). This high willingness may be related to the fact that they have a higher risk of developing cancer, pay more attention to their reproductive health, and can fully understand HPV and its vaccine through medical treatment and other means (27), for example, knowing the scientific basis for and the actual effects of vaccination even after infection. Our result also showed that willingness was significantly higher among women aged ≤45 years compared with those >45 years, possibly related to the upper age limit for vaccination and greater health awareness in the younger group (25, 55). In our sample, the HPV vaccination proportion for <45 year-group patients was 40.21%, substantially exceeding the 2.02% reported in the same region between 2018 and 2021 (2), as well as rates observed in the general population across other parts of China (66, 70), this higher uptake may reflect CIN patients’ heightened health consciousness and urgency to prevent disease recurrence. In the >45-year group, vaccination willingness remained high at 73.96%, indicating substantial interest despite age-related eligibility restrictions. This finding aligns with a recent study from Shenzhen, which reported that 47.9% of women aged 46–64 wished to extend the upper age limit for HPV vaccination, reflecting strong motivation among older women (25). However, our findings also revealed a clear gap still exists between the high willingness rate and the actual vaccination proportion, which may be attributed to economic burden, low awareness, and insufficient medical resources, underscoring the need for targeted interventions in this high-risk group (71). Due to age restrictions for current vaccination, we observed that especially the previous proportion vaccinated among women >45 years old is only 3.55%, which may increase their risk of disease recurrence and progression. We found that vaccination proportion was highest among women aged <30 years, likely reflecting policies promoting early vaccination. Among women aged 30–40 years, we observed vaccination proportion declined temporarily before rebounding. This rebound may be potentially due to higher frequencies of gynecological screening, increased health awareness, and greater motivation for catch-up vaccination to prevent recurrence (72). Previous studies have shown that screening rates are higher among women aged 35–49 years, which may provide more opportunities for screening-related health consultations, increase exposure to vaccine-related information, and ultimately improve vaccination uptake (73). Prior evidence suggests that women aged 35–64 years constitute the major group for cervical cancer incidence in China, and the elevated disease burden in this age range further underscores the importance of timely prevention (97, 98). Therefore, it is recommended to evaluate the clinical efficacy of HPV vaccination in individuals over 45 years of age and expand the appropriate age range for HPV vaccination (29).

### 4.2 HPV and its KAP among CIN patients

The present study found that the KAP scores of the willingness group were significantly higher than those of the unwillingness group. Regarding knowledge, for the item “Can the HPV vaccine prevent cervical cancer?,” The scores in the willingness group were significantly higher than those in the unwillingness group, consistent with the results of a previous study (29). But for the item “Can people who are infected with HPV also receive the HPV vaccine?,” Although the scores in the willingness group were significantly higher than those in the unwillingness group, the percentage of correct scores was still not high (49.15%). This may be because patients have misunderstandings about the indications and mechanism of action of the HPV vaccine. Such misunderstandings are deeply rooted in the delayed updating of information and limited access to information for some patients in the low-resources areas (74). Therefore, it is crucial to emphasize the message “HPV vaccination is still available after HPV infection” in health interventions. In terms of attitudes and practices, our study showed that the willingness group demonstrated higher health literacy, with a higher level of proactivity in understanding the treatment of HPV infection, motivation to cooperate with post-treatment review, and optimism about prognosis.

### 4.3 Factors influencing HPV vaccination willingness in CIN patients

Consistent with Lin’s and Wang’s studies (75, 76), our findings also showed that older age was associated with lower willingness to receive HPV vaccination among CIN patients, possibly due to limited awareness of its preventive effect on CIN progression and perceived low personal risk among CIN patients. We observed that patients’ gross monthly household income, education level, and residence were influential factors in vaccination willingness, which are consistent with other studies (77, 78). In addition, our regression model showed that sexual behavioral factors (monthly sexual intercourse frequencies and number of all sexual partners) also significantly influenced vaccination willingness. According to the 2024 China Statistical Yearbook (79), the rural poor in Gansu Province have lower incomes (average disposable income of about US$1,790.92/year; significantly lower than the national average), poor access to education and health care may cause low levels of KAP about HPV infection/vaccine, face higher risks of progression of cervical precancerous lesions and the economic burden of the disease, and are often unable to afford higher-cost vaccines. And therefore their willingness to be vaccinated is generally lower (80, 81). Therefore, vaccine prices can be negotiated with merchants at the national level and lowered through bulk wholesale purchases, upfront research and vaccination investments, or accelerated domestic vaccine development (82). Vaccination programs adopting single-dose schedules of lower-cost bivalent or quadrivalent vaccines may also be considered for high-risk groups in low-resource settings (71). In our study, urban patients exhibited higher vaccine willingness due to greater accessibility to health education. Based on our data, multiple sexual partners and frequent sex (high-risk sexual behaviors) increased the risk of HPV infection, and that patients with precancerous lesions had a higher prevalence of fear of disease recurrence or progression, and that patients with high-risk sexual behaviors were willing to be vaccinated because of the perceived increased risk of their own infection (83, 84). Other studies have similarly reported higher willingness among individuals with high-risk sexual behaviors (85, 86). In contrast, low-income individuals with low educational attainment may have negative attitudes toward the HPV vaccine due to the presence of social stigma and the perception that they are at lower risk of reinfection in our investigation (87–89).

Our parallel mediation analysis demonstrated that attitudes and practice can independently mediate the effect of knowledge on vaccination willingness. Greater knowledge enhances patients’ understanding of disease severity, threat levels, and vaccine benefits, thereby fostering positive attitudes that directly influence vaccination willingness. Knowledge also improves preventive practice, which directly impacts vaccination willingness (40, 56). Patients can further strengthen their motivation to vaccinate through proactive actions such as seeking information about HPV treatment options and encouraging regular screening. Moreover, the chain mediation model also reveals the transmission mechanism whereby knowledge influences practice through attitude, which in turn affects willingness. Enhancing knowledge may therefore promote vaccination willingness by fostering positive attitudes and proactive behaviors. These findings are consistent with previous studies (90–92). In the future, the HPV vaccine health education program should be combined with sexual life guidance, pay more attention to patients with low education, the poor, and rural patients, and focus on improving patients’ knowledge of HPV and its vaccine. This can be achieved by providing accurate, concise, and easy-to-understand health education content, as well as providing certain financial subsidies and implementing vaccine immunization programs.

Compared to the total population, our study found that in patients aged >45 years, age, education, household income, and residence remained significant predictors of HPV vaccination willingness, whereas sexual behavior was no longer significant. But disease severity became a key determinant. We observed that patients with LSIL were more willing to vaccinate than those with HSIL, likely due to greater trust in the vaccine’s preventive effect, while HSIL patients may prioritize clinical or surgical treatment (93). This difference may also relate to the natural history of CIN. LSIL has a high likelihood of spontaneous regression, with up to 90% possibly resolving within 24 months (9), whereas untreated HSIL may progress to invasive cancer in more than 30% of cases (10), prompting HSIL patients to focus more on definitive clinical management. Younger patients’ willingness is more influenced by sexual behavior, whereas older patients are influenced mainly by disease status and socioeconomic factors. Moreover, knowledge level showed no significant effect on vaccination willingness of patients aged >45 years. This may be because older patients often have relatively fixed perceptions of HPV and cervical cancer, and their willingness is more likely driven by perceived benefits and actual behaviors rather than by additional knowledge acquisition (94). Targeted interventions should emphasize sexual health for younger patients and the vaccine’s preventive effect against recurrence of high-grade lesions for older patients.

### 4.4 Reasons for willingness and unwillingness to receive HPV vaccine

Our study showed that the main reasons for patients’ willingness to receive HPV vaccination were prevention of cervical cancer (93.22%), fear of recurrence or progression (41.69%), and concern about infecting others (29.04%). This may be because of the high incidence of fear of recurrence or progression of the disease among patients with precancerous conditions, which HPV vaccination may alleviate their disease-related and psychological burden (83, 84). The main reasons for patients’ reluctance to be vaccinated were lack of knowledge (44.92%), believing they were over-age (42.52%), and fear of the vaccine being useless after already being infected (25.20%). According to our results, these might be due to insufficient knowledge and the late introduction of the vaccine, which could result in missed vaccination opportunities (95). In addition, healthcare providers play an important role in recommending HPV vaccination and promotion, and are an important source of HPV-related health information for patients. There were even patients who were unwilling to be vaccinated due to local doctors’ recommendations against it (3.94%), suggesting the need to improve healthcare workers’ related awareness and willingness to recommend (96). Therefore, the health sector needs to strengthen the training of healthcare workers in health resource-poor areas around the world in HPV-related expertise in order to provide more accurate and reliable vaccination advice to patients.

### 4.5 Innovations and limitations

This study has the following strengths: It is based on CIN patients in low-resource settings, presenting a relatively larger sample size. In addition, by using mediation models, we examined the indirect and direct effects of knowledge, attitude, and practice on HPV vaccination willingness, revealing the underlying pathways through which KAP influences vaccination willingness. Moreover, we designed KAP entries that meet the characteristics of the patient population and constructed a universal tool for assessing willingness to vaccinate, which not only provides a scientific basis for the application of HPV vaccine in the population with precancerous cervical lesions in low-health-resource areas, but also serves as a reference for the framework of the study of willingness to vaccinate in other high-risk populations and for the assessment of the vaccine rollout in low-resource settings. However, the use of convenience sampling in this study is subject to selection bias. The cross-sectional study design does not allow for the establishment of causality. In addition, actual vaccination behavior was not incorporated into the mediation analysis due to data structure constraints, which represents a methodological limitation. Finally, the inclusion of sensitive private information such as sexual behaviors may lead to reporting bias, which may affect the interpretability of the model. Therefore, we reduced the bias by guaranteeing the anonymity of the questionnaire completion and the privacy of the survey setting.

## 5 Conclusion

HPV vaccination willingness is relatively high among CIN patients in low-resource settings, yet a substantial gap exists between willingness and actual vaccination behavior. This study contributes to the field by integrating the KAP framework with mediation models to systematically explore the pathways through which knowledge, attitude, and practice influence vaccination willingness. The findings highlight that patients’ age, socioeconomic status, education, geographic region, sexual behavior, and awareness level significantly affect vaccination willingness, emphasizing the importance of targeted interventions for high-risk groups, particularly older patients with high-grade lesions, to prevent recurrence. Patients were mainly motivated by prevention of cervical cancer and fear of recurrence. The proposed mediation model provides a valuable framework for understanding vaccination behavior in low-resource settings and can inform evidence-based vaccine promotion strategies. Future research should adopt multicenter, large-sample designs to enhance representativeness and account for local cultural contexts, thereby refining intervention approaches.

## Data Availability

The datasets generated and/or analyzed during the current study are not publicly available in order to protect patient privacy, but may be made available from the corresponding author upon reasonable request.
