# Supplementary material for: HPV vaccination willingness and behavior among patients with cervical intraepithelial neoplasia in low-resource areas of Western China: a cross-sectional study
Source: Front Public Health. 2026 Jan 22;13:1708917. doi: 10.3389/fpubh.2025.1708917 (PMC12872753; doi:10.3389/fpubh.2025.1708917)
Supplement: Supplementary file 1 [file Data_Sheet_1.DOCX]

**Additional file S1**

**Survey Questionnaire on Knowledge, Attitudes, Practices, and Willingness to Receive the HPV Vaccine among Cervical Intraepithelial Neoplasia (CIN) Patients, and Informed Consent Form**

**Institution Name**: **Lanzhou University & Maternal and Child Health Hospital**

**Date of Survey: January 2024**

### ****Selection 1: Introduction****

Dear Participant,

We are conducting a research study on HPV infection awareness and willingness to receive the Human Papilloma Virus (HPV) vaccine. The purpose of this study is to gain a better understanding of patients' knowledge, attitudes, and practice (KAP) regarding HPV vaccination and infection, which will help guide future healthcare interventions.

This study was conducted in accordance with the Declaration of Helsinki. Ethical approval was obtained from the Ethics Committee of Gansu Maternal and Child Health Hospital (Approval No.: GSFY Ethics [95], September 25th, 2023).

Your participation in this survey is **completely voluntary**. The questionnaire will take approximately **20-30 **minutes**** to complete. All responses will be **strictly confidential** and used **solely for academic research**. No personally identifiable information will be collected, and your answers will not affect your medical treatment in any way.

If you agree to participate, please carefully read and sign the informed consent form before proceeding with the questionnaire.

Thank you for your time and valuable input!

All medical staff from **the School of Public Health, Lanzhou University & the Cervical Cancer Prevention and Treatment Center**, Gansu Provincial Maternal and Child Health Care Hospital.

## ****Selection 2: Patient Informed Consent Form****

**Study Title:** A Study on the Knowledge, Attitudes, Practice (KAP) and Willingness to Receive the HPV Vaccine among Patients with Cervical Precancerous Lesions

**Research Institution:** the School of Public Health, Lanzhou University & the Cervical Cancer Prevention and Treatment Center, Gansu Provincial Maternal and Child Health Care Hospital.

**Principal Investigator:** Kefan Jiang, Yawen Shao, Huiling Wang, Shumei Tuo, Ru lin

### ****Background and Purpose****

You are invited to participate in this study, which aims to assess KAP toward HPV infection and HPV vaccination. The results will be used to help improve public health strategies and vaccination promotion among patients with cervical precancerous lesions.

### ****Study Procedures****

If you choose to participate, you will be asked to complete a questionnaire regarding your knowledge and perceptions of HPV and the HPV vaccine. The survey consists of **three parts (** General social demographic information; HPV and its vaccine KAP scale; Willingness and current status of HPV vaccination **)** including **68 entries** and will take approximately **20-30 **minutes**** to complete. This study does **not** involve any medical interventions or treatments.

### ****Potential Risks and Benefits****

There are **no known risks** associated with participating in this study. While there are no direct medical benefits for participants, your responses will contribute to a better understanding of HPV vaccine awareness, which may help improve healthcare strategies for other patients in the future.

### ****Confidentiality****

All information you provide will remain **strictly confidential**. Your answers will be anonymized and analyzed only in aggregate form. Research findings may be published in academic journals, but no personally identifiable information will be disclosed.

### ****Voluntary Participation****

Your participation in this study is **entirely voluntary**. You have the right to withdraw at any time without providing a reason, and your decision will not impact your medical care or any other services you receive.

### ****Contact Information****

If you have any questions about this study, please feel free to contact:

**Principal Investigator:** Kefan Jiang
**Contact Information:** [jiangkf2023@lzu.edu.cn](mailto:jiangkf2023@lzu.edu.cn) (**E-mail** ) / +86 19502147867 (Tel.)

### ****Participant Consent****

I have read and understood the information provided above. I understand that my participation is voluntary and that I can withdraw at any time. I agree to participate in this study.

**Participant Name:**

**Participant ID:**
**Participant **S**ignature:**

**Researcher Name :**
**Researcher **Signature:****
**Survey **Dat**e:**

## ****Selection 3:**** Survey Questionnaire Questions

Ⅰ. General social demographic information Questionnaire

**The following questions are related to your basic information. Please fill in the specific details in the underscored sections, and please** make your selections based on your actual situation and mark '√' next to the corresponding option.

1. Your date of birth (Age) : year month
2. Your residence:

(1) city

(2) countryside

3. Your ethnicity:

(1) Han

(2) Ethnic Minority

4. Do you have any religions:

(1) Yes

(2) No

5. Your education level:

(1) Primary school and lower

(2) junior high school

(3) high school or secondary school education or higher

6. Your marital status?

(1) Single

(2) Married

(3) Divorced and Widowed

7. Your occupation:

(1) Unemployed

(2) Farmer

(3) Others

8. Your Healthcare payment methods:

(1) Urban and Rural Resident Basic Medical Insurance

(2) Employee Basic Medical Insurance

(3) Commercial Health Insurance

(4) Self-payment

9. Your total monthly household income, (RMB yuan)

(1) Less than 3000 yuan

(2) 3001–5000 yuan

(3) 5,000 and above

10. Do you smoke now?

(1) Yes

(2) No

11. Have you consumed alcohol in the past year?

(1) Yes

(2) No

12. What was your age at first sexual intercourse?

(1) <15 years

(2) 15-20 years

(3) 20-25 years

(4) ≥25 years

13. What is your sexual intercourse frequency (times/month) ?

(1) ≤4

(2) >4

14. What is your 6-month partner count (persons)?

(1) 0

(2) 1 partners

(3) 2 and more

15. What is the number of all sexual partners (persons) you have had until now?

(1) 1

(2) 2

(3) 3 and above

16. Do you use condoms during sexual intercourse?

(1) Always (Each time or often)

(2) Occasionally (10%)

(3) Never

17. Do you use contraceptives?

(1) Always ( Each time or often)

(2) Occasionally (10%)

(3) Never

18. Your frequency of pregnancies:

19. Your number of deliveries:

20. What is your disease severity?

(1) LSIL

(2) HSIL

21. Do you have a history of frequent gynecological infections (≥3 times/year)?

(1) Yes

(2) No

1. Do you have cervical cancer family history?

(1) Yes

(2) No

II. Knowledge, Attitude, and Practice (KAP) Scale

Instructions: The following questions are related to your knowledge, attitude, and behavior regarding HPV infection and HPV vaccination. Please make your selections based on your current situation and mark '√' next to the corresponding option.

The knowledge dimension covered six domains: HPV infection and progression (K1-K6), transmission routes (K7-K10), infection risk factors (K11-K13), vaccine-preventable diseases (K14-K16), vaccination eligibility and limitations (K17-K23), and cervical cancer screening and prevention (K24-K26). The attitude dimension included perceptions regarding HPV infection or disease recurrence (A1-A6) and vaccine-related attitudes (A7-A12). The practice dimension encompassed proactive health-seeking behaviors (P1-P2) and HPV vaccine recommendation behaviors (P3-P4).

| Knowledge Scale (K1-K26) | | | |
| --- | --- | --- | --- |
| Entries | Options | | |
| 1. HPV is a common virus | Yes | Not sure | No |
| 1. Most HPV infections are asymptomatic and resolve spontaneously within 1~2 years. | Yes | Not sure | No |
| 1. HPV infection can be classified as low-risk and high-risk. | Yes | Not sure | No |
| 4. Can you get infected with other types of HPV after being infected with one type?? | Yes | Not sure | No |
| 5. Persistent infection with high-risk HPV can lead to cervical cancer. | Yes | Not sure | No |
| 6. Some types of HPV can also cause genital warts, vulvar cancer, vaginal cancer, penile cancer, anal cancer, oropharyngeal cancer, and other diseases. | Yes | Not sure | No |
| 7. HPV can infect both women and men. | Yes | Not sure | No |
| 8. Can HPV be transmitted through sexual intercourse? | Yes | Not sure | No |
| 9. Can pregnant women infected with HPV transmit the virus to their fetuses? | Yes | Not sure | No |
| 10. Can HPV be transmitted through other means (e.g., kissing, sharing toilets or towels, eating)? | Yes | Not sure | No |
| 11. Does the chance of HPV infection increase with the number of sexual partners? | Yes | Not sure | No |
| 1. The correct use of condoms can effectively prevent HPV infection. | Yes | Not sure | No |
| 13. Does smoking increase the risk of cervical cancer? | Yes | Not sure | No |
| 14. Can HPV vaccine effectively prevent cervical cancer? | Yes | Not sure | No |
| 15. Can the HPV vaccine effectively prevent cervical cancer? | Yes | Not sure | No |
| 16. Can the HPV vaccine effectively prevent vulvar cancer, vaginal cancer, anal cancer, penile cancer, and oral cancer? | Yes | Not sure | No |
| 17. Does the nonavalent vaccine cover more types of HPV than the bivalent and quadrivalent vaccines? | Yes | Not sure | No |
| 18. Is the recommended age for HPV vaccination 9–45 years old? | Yes | Not sure | No |
| 19. Is it best to receive the HPV vaccine before becoming sexually active? | Yes | Not sure | No |
| 20. Can people who have had sexual intercourse receive the HPV vaccine? | Yes | Not sure | No |
| 21. The HPV vaccine cannot prevent all types of HPV infections. | Yes | Not sure | No |
| 22. Is it still necessary to undergo regular cervical cancer screening after receiving the HPV vaccine? | Yes | Not sure | No |
| 23. Is the recommended age for HPV vaccination 9–45 years old? | Yes | Not sure | No |
| 24. Can cervical cancer screening help detect, diagnose, and treat cervical cancer early? | Yes | Not sure | No |
| 25. Is HPV testing one of the main screening methods for cervical cancer?一 | Yes | Not sure | No |
| 26. Can actively treating chronic cervical inflammation and early cervical lesions help prevent cervical cancer? | Yes | Not sure | No |
| Attitude Scale (A1-A12) | | | |
| 1. Are you worried about getting infected with HPV again after being cured? | Yes | Not sure | No |
| 1. Are you worried about your disease coming back after treatment? | Yes | Not sure | No |
| 1. Are you willing to participate in activities related to HPV infection prevention (e.g., HPV prevention lectures)? | Yes | Not sure | No |
| 1. Do you think HPV infection can be cured? | Yes | Not sure | No |
| 1. After treatment, would you be willing to have regular HPV testing to screen for cervical cancer? | Yes | Not sure | No |
| 1. Do you want to learn more about HPV infection? | Yes | Not sure | No |
| 1. After receiving the HPV vaccine, is it still necessary to use protection during sexual intercourse? | Yes | Not sure | No |
| 1. Do you think the protection provided by the HPV vaccine (effectiveness) lasts a long time? | Yes | Not sure | No |
| 1. Do you think all eligible females must receive the HPV vaccine? | Yes | Not sure | No |
| 1. Do you think all eligible males must receive the HPV vaccine? | Yes | Not sure | No |
| 1. Have you looked into the cost and procedure of HPV vaccination? | Yes | Not sure | No |
| 1. Do you want to learn more about the HPV vaccine? | Yes | Not sure | No |
| Practice Scale (P1-P4) | | | |
| 1. Would you be proactive in learning about treatment for HPV infection? | Yes | Not sure | No |
| 1. Would you encourage your family and friends to regularly undergo cervical cancer screening? | Yes | Not sure | No |
| 1. Would you recommend the HPV vaccine to your friends? | Yes | Not sure | No |
| 1. Would you recommend the HPV vaccine to your family? | Yes | Not sure | No |

Ⅲ.Willingness and current status of HPV vaccination Questionnaire

Instructions: The following questions are related to your willingness to receive the HPV vaccine and your current vaccination status. Please select the option that best reflects your situation and mark ' √ ' next to the corresponding option.

1. Are you willing to be vaccinated against HPV?
2. Willing

(2) Not willing

1. Have you been vaccinated against HPV?

(1) Yes

(2) No

3. What are the reasons why you are willing to receive HPV vaccination?

(1) Preventing cervical cancer and preventing the disease from worsening

(2) You are already infected with HPV and worried about recurrence after treatment

(3) You are worried about your partners or loved ones being infected with HPV-related diseases

(4) The vaccine is safe

(5) Physician Advocacy Influence

(6) Vaccines are very effective.

(7) HPV vaccination for family and friends or highly recommended

(8) HPV awareness-raising activities at community health centers and other institutions

4. What are the reasons why you are not willing to receive HPV vaccination?

(1) Not knowing about HPV and HPV vaccine

(2) You are beyond the age of vaccination

(3) You have already been infected with HPV and worrying about the lack of usefulness in the follow-up

(4) Vaccines are expensive

(5) Concern about the safety of the vaccine

(6) Concern about the side effects of vaccines

(7) Concern about the effectiveness of vaccines

(8) Difficulty in making vaccine appointments

(9) The local doctor said it's not useful to inoculate against the infection.

(10) Family members do not support their own vaccination

(11) Cumbersome vaccination procedures

(12) Reproductive needs
