# Supplementary material for: HPV vaccination willingness and behavior among patients with cervical intraepithelial neoplasia in low-resource areas of Western China: a cross-sectional study
Source: Front Public Health. 2026 Jan 22;13:1708917. doi: 10.3389/fpubh.2025.1708917 (PMC12872753; doi:10.3389/fpubh.2025.1708917)
Supplement: Supplementary file 2 [file Data_Sheet_2.DOCX]

**Additional file S2**

**Supplementary R code for mediation analysis of KAP on HPV vaccination willingness**

**# Load necessary packages**

**library(lavaan)**

**library(dplyr)**

**library(mediation)**

**# Load data**

**data <- read.csv('D:/Rmyprojects/SCI/path.csv')**

**# Data preprocessing**

**df <- data %>%**

**mutate(**

**HPV_bin = as.numeric(factor(HPV.Willingness, levels = c(0,1))) - 1,**

**education = factor(education),**

**residence = factor(residence, levels = c(0,1), labels = c("Rural","Urban")),**

**income = factor(income),**

**sexual.frencency = factor(sexual.frencency, levels = c("Low","High")),**

**sexual.partner = factor(sexual.partner, levels = c("1preson","2presons","3presons"),**

**labels = c("One","Two","Three")),**

**# Dummy variables**

**edu_Junior = as.numeric(education=="Junior"),**

**edu_High = as.numeric(education=="High"),**

**income_Middle = as.numeric(income=="Middle"),**

**income_High = as.numeric(income=="High"),**

**partner_Two = as.numeric(sexual.partner=="Two"),**

**partner_Three = as.numeric(sexual.partner=="Three")**

**)**

**# ----------------------------**

**# 1. Single mediation: Attitude**

**# ----------------------------**

**med.fit1 <- lm(Attitude ~ Knowledge + age + edu_Junior + edu_High +**

**residence + income_Middle + income_High + sexual.frencency +**

**partner_Two + partner_Three, data = df)**

**out.fit1 <- glm(HPV_bin ~ Knowledge + Attitude + age + edu_Junior + edu_High +**

**residence + income_Middle + income_High + sexual.frencency +**

**partner_Two + partner_Three,**

**family = binomial(link="logit"), data = df)**

**med.out1 <- mediate(med.fit1, out.fit1, treat="Knowledge", mediator="Attitude",**

**boot=TRUE, sims=5000)**

**summary(med.out1)**

**# ----------------------------**

**# 2. Single mediation: Practice**

**# ----------------------------**

**med.fit2 <- lm(Practice ~ Knowledge + age + edu_Junior + edu_High +**

**residence + income_Middle + income_High + sexual.frencency +**

**partner_Two + partner_Three, data = df)**

**out.fit2 <- glm(HPV_bin ~ Knowledge + Practice + age + edu_Junior + edu_High +**

**residence + income_Middle + income_High + sexual.frencency +**

**partner_Two + partner_Three,**

**family = binomial(link="logit"), data = df)**

**med.out2 <- mediate(med.fit2, out.fit2, treat="Knowledge", mediator="Practice",**

**boot=TRUE, sims=5000)**

**summary(med.out2)**

**# ----------------------------**

**# 3. Parallel mediation: Attitude + Practice**

**# ----------------------------**

**med.fitA <- lm(Attitude ~ Knowledge + age + edu_Junior + edu_High +**

**residence + income_Middle + income_High + sexual.frencency +**

**partner_Two + partner_Three, data = df)**

**med.fitP <- lm(Practice ~ Knowledge + age + edu_Junior + edu_High +**

**residence + income_Middle + income_High + sexual.frencency +**

**partner_Two + partner_Three, data = df)**

**out.fit <- glm(HPV_bin ~ Knowledge + Attitude + Practice + age + edu_Junior + edu_High +**

**residence + income_Middle + income_High + sexual.frencency +**

**partner_Two + partner_Three,**

**family = binomial(link="logit"), data = df)**

**med.outA <- mediate(med.fitA, out.fit, treat="Knowledge", mediator="Attitude",**

**boot=TRUE, sims=5000)**

**med.outP <- mediate(med.fitP, out.fit, treat="Knowledge", mediator="Practice",**

**boot=TRUE, sims=5000)**

**summary(med.outA)**

**summary(med.outP)**
