# Supplementary material for: HPV vaccination willingness and behavior among patients with cervical intraepithelial neoplasia in low-resource areas of Western China: a cross-sectional study
Source: Front Public Health. 2026 Jan 22;13:1708917. doi: 10.3389/fpubh.2025.1708917 (PMC12872753; doi:10.3389/fpubh.2025.1708917)
Supplement: Supplementary file 3 [file Table_1.DOCX]

**Table S1:** Mediation Analysis Results for KAP on HPV Vaccination Willingness

| Mediated pathway | Effect Type | Estimate | 95%CI Lower | 95%CI Upper | *p*-value | Significance | Proportion Mediation |
| --- | --- | --- | --- | --- | --- | --- | --- |
| Results of the parallel mediation model: Knowledge→Attitude/Practice→HPV(Figure 3A) | | | | | | | |
| Knowledge→Attitude→HPV vaccination willingness | ACME  (Indirect) | 3.7924×10⁻⁵ | 9.668×10⁻⁸ | 4.5081×10⁻³ | <0.001 | *** | 68.37% |
|  | ADE  (Direct) | 1.7548×10⁻⁵ | -2.7547×10⁻⁴ | 3.1759×10⁻⁴ | 0.124 | ns | – |
| Knowledge→Practice→HPV vaccination willingness | ACME  (Indirect) | 2.7404×10⁻³ | 3.9596×10⁻⁶ | 1.0976×10⁻² | <0.001 | *** | 50.99% |
|  | ADE  (Direct) | 2.6342×10⁻³ | -2.0891×10⁻³ | 5.4667×10⁻³ | 0.1336 | ns | – |
| Overall | Total Effect | 5.5472×10⁻⁵+5.3746×10⁻³ | – | – | <0.001 | *** | – |
| Results of the chain mediation model: Knowledge→Attitude→Practice→HPV(Figure 3B) | | | | | | | |
| Knowledge→Attitude→Practice→ HPV vaccination willingness | ACME  (Indirect) | 1.4623×10⁻⁷ | 3.1921×10⁻¹⁰ | 1.4156×10⁻⁵ | <0.001 | *** | 31.63% |
|  | ADE  (Direct) | 3.1614×10⁻⁷ | 9.4374×10⁻¹⁰ | 1.8940×10⁻⁵ | <0.001 | *** | – |
|  | Total Effect | 4.6237×10⁻⁷ | 1.2592×10⁻⁹ | 3.3092×10⁻⁵ | <0.001 | *** | – |

Note: ACME=Average Causal Mediation Effect (indirect effect); ADE = Average Direct Effect; Total Effect (Indirect + Direct); *p*<0.05, ** *p*<0.01; ns = not significant.
