# Supplementary material for: HPV vaccination willingness and behavior among patients with cervical intraepithelial neoplasia in low-resource areas of Western China: a cross-sectional study
Source: Front Public Health. 2026 Jan 22;13:1708917. doi: 10.3389/fpubh.2025.1708917 (PMC12872753; doi:10.3389/fpubh.2025.1708917)
Supplement: Supplementary file 4 [file Table_2.DOCX]

**Table S2**: Baseline demographic and behavioral characteristics of CIN patients of >45-year-groupand univariable analysis of HPV vaccination willingness.

| Demographic information | N=338 | Willingness to vaccinate, M(IQR)/N (%) | | *Z/χ^2^* | *p* |
| --- | --- | --- | --- | --- | --- |
|  |  | Willingness group  N=250 | Unwillingness group  N=88 |  |  |
| Age, (years) | 52(IQR: 49–55.25) | 52(IQR: 48–55) | 53(IQR: 51–56.75) | -3.126 | 0.002 |
| Ethnicity |  |  |  | 0.030 | 0.863 |
| Han | 320(94.67) | 237(94.80) | 83(94.32) |  |  |
| Ethnic Minority | 18(5.33) | 13(5.68) | 5(5.20) |  |  |
| Residence |  |  |  | 50.572 | <0.001 |
| City | 197(58.28) | 174(69.60) | 23(26.14) |  |  |
| Countryside | 141(41.72) | 76(30.40) | 65(73.86) |  |  |
| Religions |  |  |  | 0.00018 | 0.989 |
| Yes | 27(7.99) | 20(8.00) | 7(7.95) |  |  |
| No | 311(92.01) | 230(92.06) | 81(92.05) |  |  |
| Educational level |  |  |  | 42.509 | <0.001 |
| Primary school and lower | 114(33.73) | 60(24.00) | 54(61.36) |  |  |
| Junior high school | 96(28.40) | 77(30.80) | 19(21.59) |  |  |
| High school or secondary school education and higher | 128(37.87) | 113(45.20) | 15(17.05) |  |  |
| Marital status |  |  |  | 0.499 | 0.480 |
| Married | 313(92.60) | 233(93.20) | 80(90.91) |  |  |
| Divorced and widowed | 25(7.40) | 17(6.80) | 8(9.09) |  |  |
| Occupation |  |  |  | 35.375 | <0.001 |
| Unemployed | 50(14.79) | 33(13.20) | 17(19.32) |  |  |
| Farmer | 132(39.06) | 78(31.20) | 54(61.36) |  |  |
| Others | 156(46.15) | 139(55.60) | 17(19.32) |  |  |
| Healthcare payment methods |  |  |  | 26.196 | <0.001 |
| Urban and Rural Resident Basic Medical Insurance | 90(26.63) | 57(22.80) | 33(37.50) |  |  |
| Employee Basic Medical Insurance | 103(30.47) | 95(38.00) | 8(9.09) |  |  |
| Self-payment | 145(42.90) | 98(39.20) | 47(53.41) |  |  |
| Total monthly household income, (yuan) |  |  |  | 46.972 | <0.001 |
| <3000 | 95(28.11) | 47(18.80) | 48(54.55) |  |  |
| 3000-5,000 | 120(35.50) | 92(36.80) | 28(31.81) |  |  |
| 5,000 and above | 123(36.39) | 111(44.40) | 12(13.64) |  |  |
| Smoking |  |  |  | 1.684 | 0.300 |
| Yes | 3(0.89) | 2(5.99) | 1(1.14) |  |  |
| No | 335(99.11) | 248(99.20) | 87(98.86) |  |  |
| Alcohol consumption |  |  |  | 0.529 | 0.467 |
| Yes | 42(12.43) | 33(13.20) | 9(10.23) |  |  |
| No | 296(87.57) | 217(86.80) | 79(89.77) |  |  |
| Age at first sexual intercourse, (years) |  |  |  | 12.752 | 0.005 |
| <15 years | 5(1.48) | 3(1.20) | 2(2.27) |  |  |
| 15–20 years | 60(17.75) | 34(13.60) | 26(29.55) |  |  |
| 20-25 | 198(58.58) | 152(60.80) | 46(52.27) |  |  |
| ≥25 | 75(22.19) | 61(24.40) | 14(15.91) |  |  |
| Sexual intercourse frequencies, (times/month) |  |  |  | 0.937 | 0.333 |
| ≤4 | 220(65.09) | 159(63.60) | 61(69.32) |  |  |
| >4 | 118(34.91) | 91(36.40) | 27(30.68) |  |  |
| 6-month partner count, (persons) |  |  |  | 0.588 | 0.745 |
| 0 | 82(24.26) | 58(23.20) | 24(27.27) |  |  |
| 1 | 252(74.56) | 189(75.60) | 63(71.59) |  |  |
| 2 and above | 4(1.18) | 3(1.20) | 1(1.14) |  |  |
| Number of all sexual partners, (persons) |  |  |  | 4.510 | 0.105 |
| 1 | 283(83.73) | 203(81.20) | 80(90.91) |  |  |
| 2 | 42(12.43) | 36(14.40) | 6(6.82) |  |  |
| 3 and above | 13(3.84) | 11(4.40) | 2(2.27) |  |  |
| Condom use |  |  |  | 20.479 | <0.001 |
| Always (each time or often) | 43(12.72) | 38(15.20) | 5(5.68) |  |  |
| occasionally | 78(23.08) | 69(27.60) | 9(10.23) |  |  |
| never | 217(64.20) | 143(57.20) | 74(84.09) |  |  |
| Contraceptive use |  |  |  | 5.392 | 0.067 |
| Always (each time or often) | 3(0.89) | 2(0.80) | 1(1.14) |  |  |
| Occasionally | 38(11.24) | 34(13.60) | 4(4.55) |  |  |
| Never | 297(87.87) | 214(85.60) | 83(94.31) |  |  |
| Frequency of pregnancies | 3(IQR: 2–4) | 3(IQR: 2–4) | 3(IQR: 2–4) | -1.586 | 0.113 |
| Number of deliveries | 1(IQR: 1–3) | 2(IQR: 1–2) | 2(IQR: 2–3) | -5.159 | <0.001 |
| Disease severity |  |  |  | 9.651 | 0.002 |
| LSIL | 243(71.89) | 191(76.40) | 52(59.09) |  |  |
| HSIL | 95(28.11) | 59(23.60) | 36(40.91) |  |  |
| History of frequent gynecological infections, (≥3times/year) |  |  |  | 3.055 | 0.080 |
| Yes | 220(65.09) | 156(62.40) | 64(72.73) |  |  |
| No | 118(34.91) | 94(37.60) | 24(27.27) |  |  |
| Cervical cancer family history |  |  |  | 1.065 | 0.302 |
| Yes | 3(0.89) | 3(1.20) | 0(0.00) |  |  |
| No | 335(99.11) | 247(98.80) | 88(100.00) |  |  |
| Knowledge score | 62(IQR: 54–70) | 66(IQR: 59–72) | 52(IQR: 52–54) | -12.278 | <0.001 |
| Attitude score | 31(IQR: 27–33) | 32(IQR: 30–34) | 23(IQR: 22–26) | -12.704 | <0.001 |
| Practice score | 11(IQR: 8–12) | 12(IQR: 10–12) | 6(IQR: 4–8) | -13.399 | <0.001 |
| Total | 338(100.00%) | 250(87.45%) | 88(12.55%) |  |  |

Notes:

Continuous variables are presented as M (IQR), where M is the median and IQR is the interquartile range (25th-75th percentiles). Categorical variables are presented as N (%), indicating frequency and percentage. Abbreviations: N, number of participants; Z, Z values for Mann-Whitney U test; χ², chi-square values for categorical variables; OR, odds ratio; 95% CI, 95% confidence interval.
