# Supplementary material for: HPV vaccination willingness and behavior among patients with cervical intraepithelial neoplasia in low-resource areas of Western China: a cross-sectional study
Source: Front Public Health. 2026 Jan 22;13:1708917. doi: 10.3389/fpubh.2025.1708917 (PMC12872753; doi:10.3389/fpubh.2025.1708917)
Supplement: Supplementary file 5 [file Table_3.DOCX]

**Table S3**. Multivariable association between HPV KAP scores and HPV vaccination willingness in the >45-year group.

| Variables | Model 1 | |  | Model 2 | |  | Model 3 | |
| --- | --- | --- | --- | --- | --- | --- | --- | --- |
|  | *OR*(95%*CI*) | *p* value |  | *OR*(95%*CI*) | *p* value |  | *OR*(95%*CI*) | *p* value |
| Knowledge | 1.053(0.938–1.183) | 0.380 |  | 1.092(0.963–1.239) | 0.170 |  | 1.149(0.964–1.369) | 0.121 |
| Attitude | 1.545(1.235–1.932) | <0.001 |  | 1.614(1.259–2.069) | <0.001 |  | 1.787(1.253–2.548) | 0.001 |
| Practice | 2.077(1.525–2.829) | <0.001 |  | 2.269(1.581–3.257) | <0.001 |  | 3.495(1.962–6.223) | <0.001 |

Notes:

Abbreviations: OR, odds ratio; CI, confidence interval.

Model 1: No covariates controlled.

Model 2: Controlled for sociodemographic variables, including age, ethnicity, current residence, religious, education level, occupation, marital status, method of medical payment, and total monthly household income.

Model 3: Controlled for all covariates, additionally including smoking, alcohol consumption, age at first sexual intercourse, frequency of sexual activity, number of sexual partners in the past six months, total number of sexual partners, condom usage frequency, oral contraceptive usage frequency, number of pregnancies, number of deliveries, disease severity, history of frequent gynecological inflammation, and family history of cervical cancer.
